# The Burden of Uterine Fibroids from the Perspective of US Women Participating in Open-Ended Interviews

**DOI:** 10.1089/whr.2021.0086

**Published:** 2022-03-04

**Authors:** Elke Hunsche, Viatcheslav Rakov, Kayla Scippa, Brooke Witherspoon, Laura McKain

**Affiliations:** ^1^ Department of Global Market Access & HEOR, Myovant Sciences GmbH, Basel, Switzerland.; ^2^ Global Market Access and Health Economics/Outcomes Research, Myovant Sciences GmbH, Basel, Switzerland.; ^3^ Endpoint Outcomes, Long Beach, California, USA.; ^4^ Endpoint Outcomes, Boston, Massachusetts, USA.; ^5^ Myovant Sciences, Inc., Brisbane, California, USA.

**Keywords:** burden, concept elicitation, interview, patient perspective, uterine fibroids

## Abstract

**Background:**

Research on women's perspective of uterine fibroids (UF) experiences using their own words is limited. This study aimed to provide new insights on the symptoms experienced and their impacts on daily life.

**Methods:**

Interview substudy in 30 US women with heavy menstrual bleeding (HMB) associated with UF who completed one of two phase 3, randomized, double-blind, placebo-controlled trials (LIBERTY 1 and 2; ClinicalTrials.gov identifiers: NCT03049735, NCT03103087). Women who consented to participate in this substudy were interviewed after their last clinical trial study visit. Concepts (*i.e.*, symptoms and impacts) of importance to women were determined *via* open-ended questions, and the frequency of symptoms and their impacts, including the relationship between pain and menstruation, were assessed. Data were analyzed using established qualitative research methods, including grounded theory and constant comparative methods, and concept saturation was assessed.

**Results:**

Fifteen unique symptoms of UF emerged: the most commonly reported were HMB (*n* = 30, 100.0%), pelvic pain (*n* = 28, 93.3%), and passing of blood clots (*n* = 24, 80.0%). In total, 25 unique impacts were identified across eight concepts: physical impacts, activities of daily living, sleep, emotional impacts, sex life, social impacts, work and school, and financial impacts. Concept saturation was achieved for both symptoms and impacts.

**Conclusion:**

This study provides data on the symptoms experienced by women with HMB associated with UF, as well as the negative impacts of these symptoms as reported using their own words. The study findings confirm the significant burden associated with symptomatic UF.

## Introduction

Uterine fibroids (UF) are a common type of benign tumor characterized by the overgrowth of connective or smooth muscle tissue in the uterus.^1,2^ UF may be asymptomatic; however, at least 25% of women experience symptoms,^3,4^ often including heavy menstrual bleeding (HMB), anemia, and pelvic pain.^1,5,6^ In addition, UF may lead to bulk-related symptoms related to the mass effect of the enlarged uterus, which include pelvic pressure, increased urinary frequency, leg and back pain, constipation, bloating, and dyspareunia.^1,5–7^

An estimated 70% of women have at least one fibroid before menopause.^2,8^ Black women are at increased risk of developing UF, compared with other racial groups.^9^ UF symptoms can lead to disruption in work productivity, sleep, and social activities,^10^ and significantly impact women's health-related quality of life.

Although evidence on UF burden exists from cross-sectional studies and surveys, little is known about women's personal experiences and perspectives on the symptoms and impacts of UF, described in their own words. Patient interviews reported here aimed to gather patient experience data through open-ended questions, rather than from a survey with predefined answering options. This study sought to provide new insights into UF burden, in terms of patient-relevant symptoms and their impacts on the lives of women with HMB associated with UF. In addition, the relationship between UF-related pain and menstruation was assessed in a *post hoc* analysis.

## Materials and Methods

### Study design and eligibility criteria

The results reported here are from an interview substudy conducted in US women who had completed the LIBERTY 1^11^ (ClinicalTrials.gov identifier: NCT03049735) or the replicate LIBERTY 2^11^ (ClinicalTrials.gov identifier: NCT03103087) trials. These were phase 3, randomized, double-blind, placebo-controlled studies that assessed the efficacy and safety of relugolix 40 mg once daily in combination with estradiol 1 mg and norethindrone acetate 0.5 mg, versus placebo, for 24 weeks in women with HMB associated with UF.^12^ All women had UF confirmed by ultrasound and objectively confirmed HMB (menstrual blood loss of ≥160 mL during one cycle or ≥80 mL per cycle for two menstrual cycles, as measured by the alkaline hematin method during the screening period).^12^

All US sites with potentially eligible patients treated in the LIBERTY studies were approached for participation in the present substudy; the US sites included were those who were interested in recruiting patients for this substudy. Thirty women from US sites, who completed their last study visit (week 24) of either LIBERTY study, who consented to participate in the interview substudy, and who were able to speak, read, write, and comprehend English were interviewed. An additional inclusion criterion was an improvement on the five-point patient global assessment (PGA) scale for symptoms by week 12. The improvement in PGA scale was required for questions related to what women considered to be a meaningful improvement on different patient-reported outcomes (the results of which will be reported elsewhere).

The interview study was submitted to the Advarra Institutional Review Board for ethics review and approval before contact with patients.

### Interviews

Interviews were conducted *via* a web/internet-based video platform or telephone by a trained interviewer and occurred 3–14 days after the last study visit (week 24). The interviews aimed to explore concepts (*i.e.*, symptoms and impacts) of importance to women with HMB associated with UF.

Interviews were based on a semistructured interview guide and included open-ended questions to encourage spontaneous responses, followed by targeted probes as needed. The interview guide included topics, questions, and probes designed to understand the overall UF burden. Women were asked to describe symptoms and impacts they experienced as a result of UF before enrollment in LIBERTY 1 or 2, taking their lifetime experience with UF into account. If not spontaneously reported, women were asked to characterize UF symptoms and impacts in terms of frequency, severity, duration, and onset. Interviewers probed only as needed to capture relevant data; no probes were included to address the relationship between symptoms and menstruation.

Each interview was audio recorded, with the woman's prior consent.

### Adverse event reporting

Although women were asked to describe UF symptoms and impacts before participating in LIBERTY 1 or 2, they could spontaneously report adverse events potentially related to the study drug during the interview. If women reported a novel symptom or worsening of an existing symptom considered related to treatment, this was reported in the clinical trials (adverse events are not presented here).

### Data analysis

#### Coding scheme and analysis process

Audio recordings of the interviews were transcribed verbatim and anonymized. A coding scheme was developed using ATLAS.ti version 8.3.20 software (Atlas.ti GmbH, Berlin), taking into account whether women reported concepts spontaneously (*i.e.*, without prompting from the interviewer) or after probing. Coding was an iterative process, so the initial code list was updated as necessary to reflect the actual terms used by women to describe concepts.

The analysis process was guided by established qualitative research methods, including grounded theory and constant comparative method.^13,14^ Unique impacts of the symptoms were identified and grouped into broader concepts, helping to identify and explain patterns and relationships within the data set.^15,16^ Ultimately, frequencies of unique concepts (symptoms and impacts, spontaneously reported and after probing) were aggregated and interpreted with accompanying example quotes.

Of note, the total number of impacts identified could be greater than the total number of women who reported the concept, because some women provided multiple examples and descriptions of how UF impacted their lives.

#### Intercoder reliability

There were a total of four coders, each of whom independently coded the first transcript. Intercoder reliability—the extent to which independent coders were concordant in coding—was evaluated using percentage agreement. Although the literature does not provide a consistent benchmark for an acceptable agreement threshold, 90% was determined *a priori* and is rigorous by the benchmarks in the literature (*i.e.*, 70%–94%).^17^ Coders met regularly throughout the coding process to ensure that concepts were coded consistently, and coding differences were reconciled as they emerged.

#### Concept saturation

Qualitative data from interviews were also assessed for concept saturation, namely the point when additional interviews were unlikely to yield new information (*i.e.*, new concepts of importance and relevance to women).^14^

To evaluate concept saturation, concepts that spontaneously emerged from interviews were analyzed in cohorts according to the order in which the data were collected. To assess whether new concepts had emerged, those reported in the first seven interviews (23.3%) were compared with those in the next eight interviews (26.7%) conducted. Both these cohorts of interviews (*n* = 15; 50.0%) were compared with the next seven interviews (23.3%), and subsequently all of these interviews (*n* = 22; 73.3%) were compared with the last eight interviews (26.7%). Achievement of saturation was used to confirm the adequacy of the sample size.

## Results

### Patient disposition

Thirty women participated in interviews across 15 US sites. Most women (*n* = 26; 86.7%) opted for phone interviews, and the remainder (*n* = 4; 13.3%) opted for web/internet-based video platform interviews.

### Participant demographics and health information

The mean age of the women was 43 years (range: 35–51 years) and 70% (*n* = 21) were Black or African American. Most (*n* = 26; 86.7%) self-reported college or higher education as their highest education level. Table 1 shows full demographic, health, and educational information.

**Table 1.** Baseline Demographic, Health, and Educational Information of Women with Heavy Menstrual Bleeding Associated with Uterine Fibroids

Baseline characteristic	Interview sample (***n*** = 30)
Age (years)	
Mean (standard deviation)	42.6 (4.6)
Range	35–51
Race	
Black or African American	21 (70.0)
White	9 (30.0)
Ethnicity	
Not Hispanic/Latina	24 (80.0)
Hispanic/Latina	6 (20.0)
Highest level of education	
High school (no degree) or less	2 (6.7)
High school graduate	2 (6.7)
Some college (no degree)	11 (36.7)
Associate's degree	4 (13.3)
Bachelor's degree	5 (16.7)
Master's degree	4 (13.3)
Other	2 (6.7)

Values are *n* (%) unless otherwise indicated.

### Intercoder reliability

Acceptable intercoder reliability (*i.e.*, 90% agreement) was achieved after four coders independently coded the first transcript.

### Concept saturation

Concept saturation was achieved for the 15 (total) spontaneously reported symptoms and the 25 (total) spontaneously reported impacts after 7 (23.3%) and 10 (33.3%) of the interviews, respectively.

### Burden of UF: UF symptoms

**FIG. 1.**
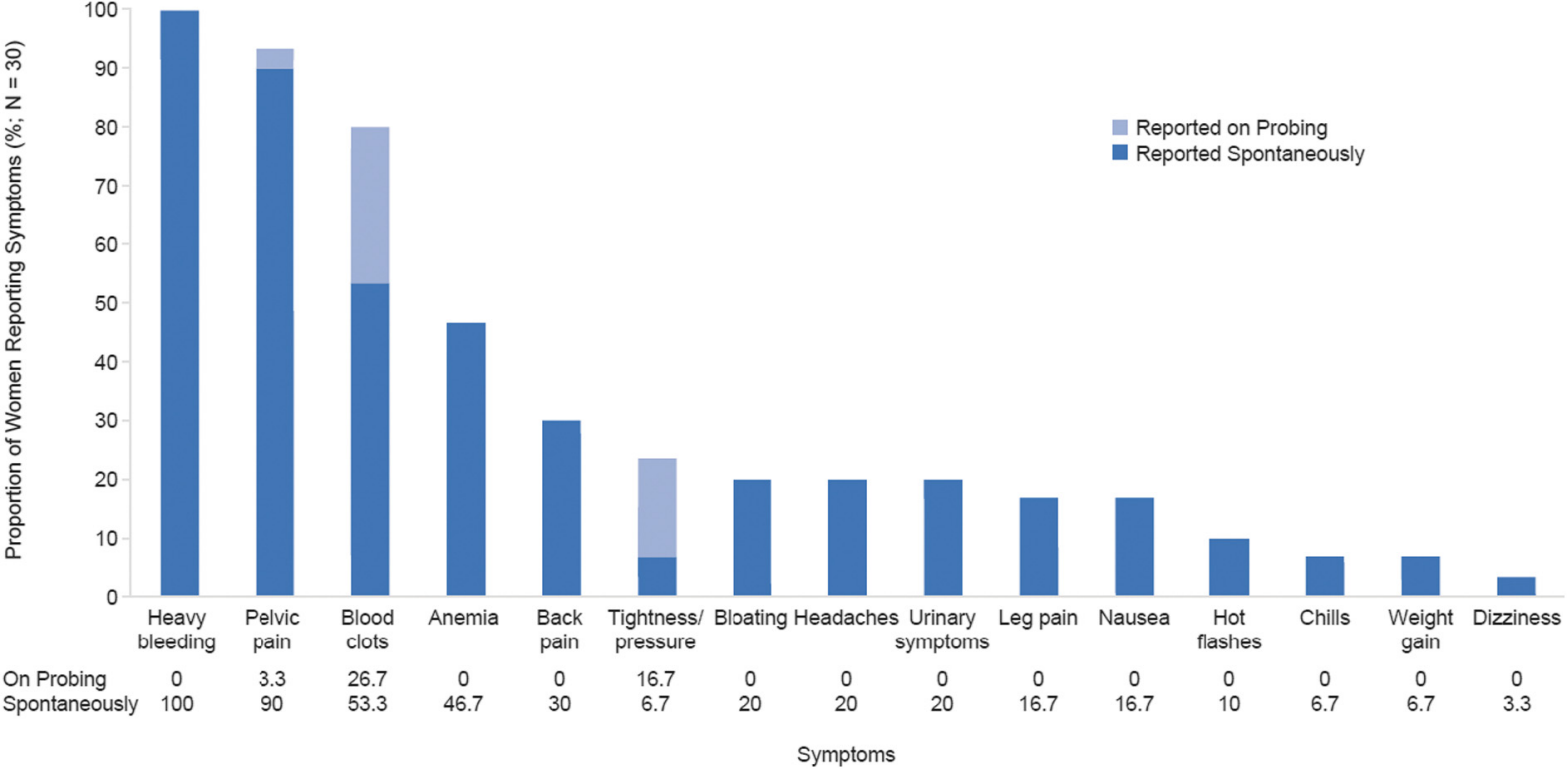
Symptoms of UF reported by women with HMB associated with UF (*n* = 30). HMB, heavy menstrual bleeding; UF, uterine fibroids.

Descriptions of 15 unique UF symptoms emerged from interviews: these were reported both spontaneously and following probing (Fig. 1). The most commonly reported symptom of UF was HMB (*n* = 30; 100.0%); one woman characterized HMB as “no matter how many sanitary towels you put in, when the blood comes out, it drains down to your feet because it pours.”

**FIG. 2.**
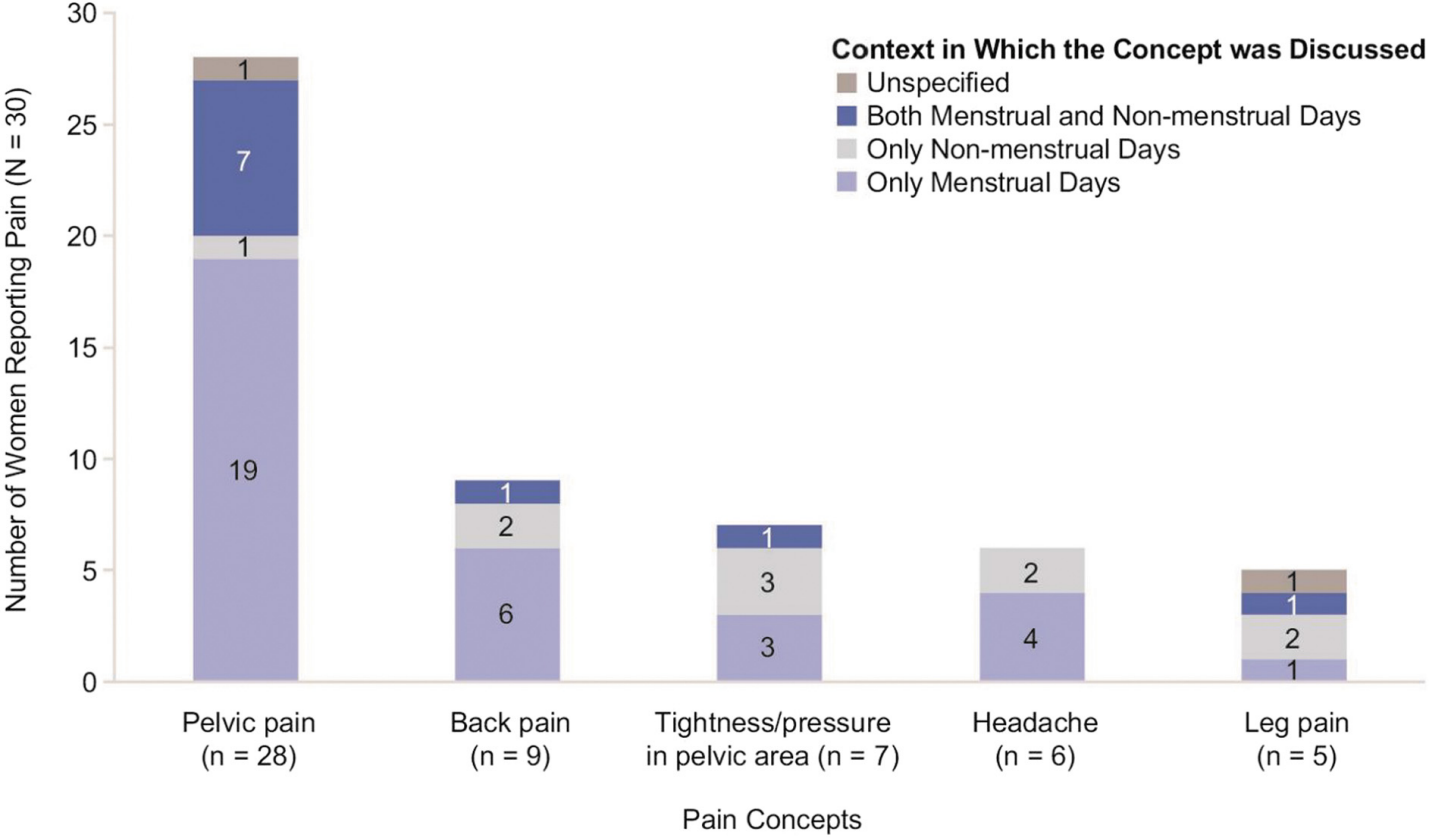
Pain concepts reported by women with HMB associated with UF, and their relationship to menstruation (*n* = 30). HMB, heavy menstrual bleeding; UF, uterine fibroids.

Pelvic pain was frequently highlighted by women (*n* = 28; 93.3%) and commonly characterized as “cramping” in the pelvic area that occurred mostly, but not always, during menstrual days; women provided descriptions such as: “It would be like a stabbing pain. I could be walking—just walking around a store and literally be broke down, like something literally is trying to rip out of me—or it's a sharp, stabbing pain.” Of women experiencing pelvic pain, most (*n* = 19 of 28; 67.8%) reported feeling pain exclusively during menstrual days, 1 (*n* = 1 of 28, 3.6%) mentioned having pelvic pain exclusively during nonmenstrual days, and 7 (*n* = 7 of 28; 25%) reported experiencing pelvic pain during both menstrual and nonmenstrual days. Figure 2 provides detailed results on whether each pain concept was reported in the context of menstrual days.

Another symptom commonly reported (*n* = 24; 80.0%) was passing of blood clots; one woman commented: “I used to have clots as big as a jellyfish, where I could just stand up at work, like I said, and they would just fall out.”

Anemia was reported by nearly half of women (*n* = 14; 46.7%) and comments included: “I was really anemic, so I was just tired all the time. All the time. … My hair was falling out a lot … my nails were kind of gross and brittle.” Back pain was mentioned by a third of women (*n* = 9; 30.0%) and was described by one woman as: “lower back pain or just like a sharp pain in my side.” Of the women experiencing back pain, most (*n* = 6 of 9; 66.7%) reported feeling pain exclusively during menstrual days, 2 (*n* = 2 of 9; 22.2%) mentioned feeling back pain exclusively during nonmenstrual days, and one woman (*n* = 1 of 9; 11.1%) reported having back pain during both menstrual and nonmenstrual days.

Seven women (23.3%) described a sensation of tightness/pressure in the pelvic area, separate from pelvic pain, which was reported to occur exclusively during menstrual days by three women (*n* = 3 of 7; 42.9%), exclusively during nonmenstrual days by three women (*n* = 3 of 7; 42.9%), and under both circumstances by one woman (*n* = 1 of 7; 14.3%). Pelvic tightness/pressure was described by one woman as: “The pressure is like a heavy object. It's like just sitting there … it's painful.”

Bloating was reported by six women (20.0%) and was described both in terms of appearance (*n* = 3 of 6; 50.0%) and sensation (*n* = 4 of 6; 66.7%); comments included: “I didn't want to look pregnant, and would always be asked when is the baby due, because of my uterus and the pressure and the bloated feeling.”

Six women (20.0%) also reported experiencing headaches due to UF: of these women, 4 (66.7%) experienced headaches only during menstrual days, and 2 (33.3%) had headaches related to UF during nonmenstrual days. Comments included: “I would get a headache. So, I would get the headache knowing it [period] was coming.” Urinary symptoms were reported by six women (20.0%), described as either urinary urgency or leakage; with comments like: “Sometimes I have to wear… pantyliners daily to support my bladder leak. And the bladder leak is only because… the biggest fibroid I have is sitting on my bladder.”

Leg pain was a less common symptom (*n* = 5; 16.7%) and reported to occur exclusively during nonmenstrual days for two women (40.0%) and one woman each (20.0%) exclusively during menstrual days, under both circumstances, and under unspecified conditions (*i.e.*, patient did not state whether leg pain occurred on menstrual days and/or nonmenstrual days). Descriptions provided included: “At night when I'm sleeping, I have to constantly rotate because of the leg pain.”

Less commonly reported symptoms (*n* ≤ 5; 16.7%) included nausea, hot flashes, chills, weight gain, and dizziness. A summary of example quotes from women about their experiences of UF symptoms is shown in Table 2.

**Table 2.** Example Quotes Regarding Uterine Fibroid Symptoms, as Reported by at Least 20% of Women with Heavy Menstrual Bleeding Associated with Uterine Fibroids (***n*** = 30)

Sign/symptom	***n*** (%)	Example patient quote
Heavy bleeding	30 (100)	*I developed just very heavy periods, where—you know, soiling my clothes—my underwear, my clothes, and my bed at night. So, I had the problem of just very heavy bleeding.*
Pelvic pain	28 (93.3)	*It would be like a stabbing pain. … like something literally is trying to rip out of me—or it's a sharp, stabbing pain. And it would be crippling.*
Blood clots	24 (80)	*I would pass really huge blood clots as well. … Maybe the size like of a 50-cent piece.*
Anemia	14 (46.7)	*But then the anemia—I was very anemic, always taking iron pills, very tired, very—sometimes I was very, very lightheaded.*
Back pain	9 (30)	*So there was no bleeding but there was still pain. Like lower back pain or just like a sharp pain in my side.*
Tightness/pressure	7 (23.3)	*Sometimes like my uterus just would feel heavy, like really heavy and uncomfortable. And there's like pressure there. … there was pressure and a heaviness in the pelvic area.*
Bloating	6 (20)	*I get really bloated when I'm on my period and I think it's worse because of the fibroids. My belly sometimes is so swollen I can only wear loose sweatpants.*
Headaches	6 (20)	*I would know it was coming oftentimes because I would get a headache. So, I would get the headache knowing it [period] was coming.*
Urinary symptoms (*i.e.*, urgency leakage)	6 (20)	*Also, I would always feel like I had to urinate, even when I didn't.*

### Burden of UF: impacts of UF symptoms

**FIG. 3.**
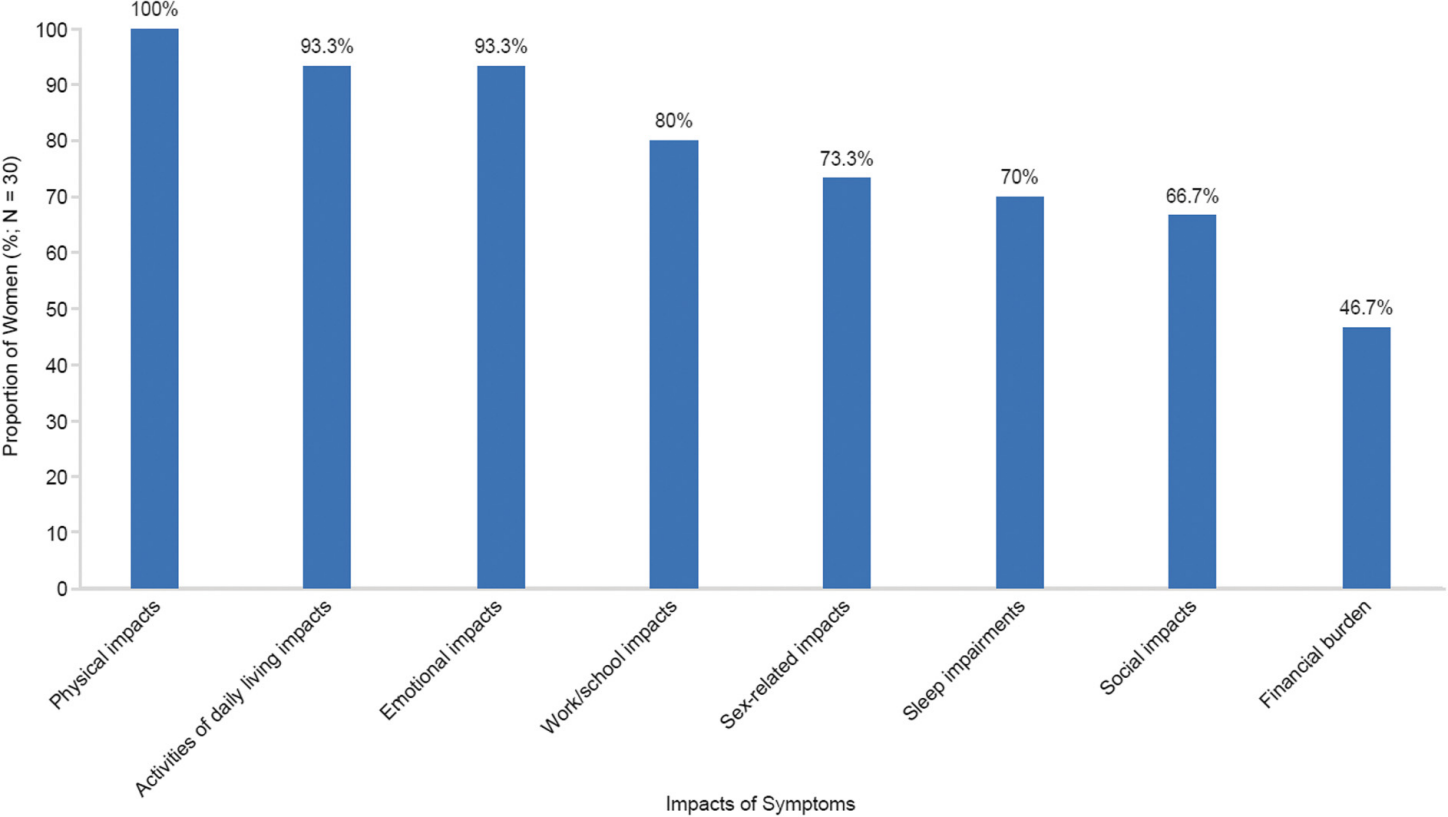
Summary of impacts of symptoms of UF as reported in interviews by women with HMB associated with UF (*n* = 30).

A total of 25 unique UF symptom impacts emerged from the interviews, across eight concepts: physical, activities of daily living, sleep, emotional, sex life, social, work/school, and financial impacts. All impacts were spontaneously reported by at least one woman (3.3%). The reported impacts of UF symptoms are summarized by overall concepts in Figure 3. A summary of example quotes for UF symptom impacts is shown in Table 3.

**Table 3.** Example Quotes Regarding the Impact of Uterine Fibroid Symptoms as Reported by at Least 20% of Women with Heavy Menstrual Bleeding Associated with Uterine Fibroids (***n*** = 30)

Impact according to concept	***n*** (%)	Example patient quote
Activities of daily living impacts		
Stopped doing daily activities	24 (80.0)	*When I had to clean the house and I was on my period, I wouldn't do it. I would just wait until the week after, because it would make me […] feel so horrible.*
Change the way daily activities are done	19 (63.3)	*When I'm on my period or when I know I'm going to have my period I have to shift my whole life around. I definitely don't plan any events during my week of hell.*
Negative impact on leisure activities	13 (43.3)	*When I'm on my period, I don't do any leisure activities. …I don't do anything fun. I just lay in my bed and wait for it to be over.*
Emotional impacts		
Feel embarrassed	14 (46.7)	*I think the biggest thing is the embarrassment surrounding it. It's really embarrassing to bleed through your clothes in public.*
Feel worried/anxious	11 (36.7)	*I am always worried that it is going to get worse and I'll be even more limited than I already am.*
Feel sad/depressed	11 (36.7)	*I would get depressed because I couldn't really do anything. I didn't understand what was going on in my body.*
Fear of bleeding through clothes	10 (33.3)	*I was afraid that I'd get blood all over myself in public and that they would notice I was bleeding.*
Feel angry/irritable	8 (26.7)	*I would be—get angry, so I'm constantly irritated. And I was always irritable. … irritable because I'm spotting, like this will not go away.*
Financial burden		
Negative impact on finances	14 (46.7)	*Financially the cost of buying a lot of pads and not just pads but Depends* ^a^ *and Poseys* ^a^ *—the Poseys and going through all that pain medication and iron tablets and vitamins—yeah, it affected me.*
Physical impacts		
Tired/fatigued	26 (86.7)	*You could get a good night's sleep, and you'll just still feel drained all throughout the day, and you'll just feel like you want to go lay down and go sleep. You're just constantly tired.*
Negative impact on physical functioning	16 (53.3)	*You can't make any kind of sudden movements. Standing up hurt. Walking hurt. Sitting down is uncomfortable.*
Negative impact on exercise	15 (50.0)	*Yeah, because of the bleeding, I cannot exercise during my period, because then the flow ends up being heavier, and I practically have to break and change. … the time I'm on my period, I'm not doing any exercise, I'm not doing any heavy lifting, I'm not doing any of those things.*
Sex-related impacts		
Negative impact on sex life	20 (66.7)	*And sex is out of the question when I'm on my period. … I'm bleeding so much that I won't let anyone near me during that time. It also has hurt in the past when I've tried so I just choose to avoid sex when I'm on my period.*
Negative impact on romantic relationships	6 (20.0)	*Yes. I would have to say I'm not in a relationship because of my uterine fibroids because I don't care to have sex because it's painful.*
Sleep impairments		
Difficulty staying asleep	21 (70.0)	*But I would have to get up in the middle of the night and change my pad because I messed up my bed. … So, my sleep is interrupted, because I'm not sleeping through the whole night, you know, because I got to go change and—change sheets, change bed, you know, all that kind of stuff.*
Social impacts		
Negative impact on social activities	20 (66.7)	*Like if I wanted to go out with family or friends, I wouldn't go, because you would be scared if you would mess up your clothes.*
Work/school impacts		
Loss of productivity or limited at work/school	15 (50.0)	*I mean before when I would go into work, I certainly wasn't as efficient. I like to think I'm good at my job. When I'm on my period, it gets in the way of me being able to do my job.*
Miss work/school	12 (40.0)	*That's what the very severe when it came to where I wouldn't be able to go to work sometimes because I be in so much pain.*

^a^ Brand names of sanitary products.

#### Physical impacts

Feeling tired, defined as a feeling of intense fatigue often occurring during the first few days of menstruation, was the most frequently reported physical impact (*n* = 26; 86.7%). Women provided statements such as: “Well, the first day I used to be in bed all the time. I couldn't stand up” and “I was just tired all the time. … Just exhausted and worn out.” Women most commonly attributed feeling tired/fatigued to anemia or heavy bleeding, with explanations like: “I was really anemic, so I was just tired all the time.”

Negative impacts on physical functioning (*n* = 16; 53.3%) and exercise (*n* = 15; 50.0%) were also reported. A negative impact on physical functioning was characterized as difficulty completing basic physical tasks (*e.g.*, walking, sitting, standing, lifting) because of UF symptoms, whereas a negative impact on exercise referred to difficulty completing an exercise regimen such as running, aerobics, cycling, or hiking.

#### Activities of daily living impacts

Twenty-four women (80.0%) reported that UF symptoms stopped them from doing their daily activities (*i.e.*, an inability to do tasks such as housework, running errands, and driving), and statements included: “As it got worse and worse, I just stopped doing things.”

Nineteen women (63.3%) said that UF symptoms changed the way they did things (*i.e.*, a modification in the method of completion of daily activities such as housework and running errands), with comments including: “I had to really just kind of plan my life around my period.” Five women (16.7%) experienced limitations of their daily activities (*e.g.*, an inability to fully complete daily activities because of UF symptoms). Thirteen women (43.3%) described a negative impact on leisure activities such as shopping or attending movies.

#### Sleep impairments

The majority of women experienced a negative impact on sleep, including difficulty staying asleep (*n* = 21; 70.0%) and falling asleep (*n* = 4; 13.3%). Waking up during the night to change feminine hygiene products (*n* = 15 of 21; 71.4%) and soiled clothing/bed linens (*n* = 6 of 21; 28.6%) were the main causes of sleep disturbance in women who had difficulty staying asleep. For example, one woman described “[I] have to get up every hour throughout the night to change my tampon and pad or else I bleed through the clothes onto the mattress.”

Women described difficulty falling asleep due to pelvic pain (*n* = 3 of 4; 75.0%), heavy bleeding (*n* = 2 of 4; 50.0%), and leg pain (*n* = 1 of 4; 25.0%), reporting for example: “[I] cannot sleep for the pain, for the bleeding. Very uncomfortable.”

#### Emotional impacts

Negative impacts on emotional well-being were reported by nearly all women (*n* = 28; 93.3%). Women most commonly expressed feeling embarrassed (*n* = 14; 46.7%), worried or anxious (*n* = 11; 36.7%), sad or depressed (*n* = 11; 36.7%), or angry or upset (*n* = 8; 26.7%) about UF symptoms. Among those worried or anxious, women indicated that they felt this way because of concerns over symptoms, with 10 women (33.3%) spontaneously reporting being afraid of bleeding through clothing; one woman said: “I think the biggest thing is the embarrassment surrounding it. It's really embarrassing to bleed through your clothes in public.” Other emotional impacts reported (*n* ≤ 5; 16.7%) included feeling self-conscious, overwhelmed, stressed, or disgusting.

#### Sex-related impacts

A negative impact on sex life and romantic relationships was reported by 20 (66.7%) and 6 (20.0%) women, respectively. One woman described the impact on her sex life as: “sex is out of the question when I'm on my period … I'm bleeding so much that I won't let anyone near me during that time. It also has hurt in the past when I've tried so I just choose to avoid sex when I'm on my period.” In addition, one woman who experienced challenges in romantic relationships recounted: “it cost me relationships with the opposite sex, because I was not—I could not be sexually active with them.”

#### Social impacts

A negative impact of UF symptoms on social activities was mentioned by 20 women (66.7%). Women described missing activities such as outings with friends: “I've missed a friend's wedding. I've missed barbecues and things—family time and things. And to me, that's very important.”

Women provided the following reasons for missing social activities: fear of embarrassment due to heavy bleeding (*n* = 6 of 20; 30.0%), severity of their symptoms (*n* = 4 of 20; 20.0%), a need to stay in bed (*n* = 3 of 20; 15.0%), inability to participate in physical activities (*n* = 2 of 20; 10.0%), the amount of planning required to participate (*n* = 2 of 20; 10.0%), and feeling negative emotions (*n* = 2 of 20; 10.0%). One woman (*n* = 1 of 20; 5.0%) also commented that although she could participate in social activities, the symptoms would “interrupt [her] activity.”

#### Work/school impacts

Loss of productivity and limitations at work or school were reported by half of women (*n* = 15; 50.0%). Specifically, eight of these women (*n* = 8 of 15; 53.3%) cited needing to interrupt their workflow to address UF symptoms (*e.g.*, frequent trips to the restroom to change pads or tampons), and eight women (*n* = 8 of 15; 53.3%) reported that they were limited in the type of work they were able to complete. For example, one woman elaborated: “I work at a warehouse, so I have to move all day. So, it'll restrict my movement. It'll restrict me how fast I can get my job done because of my uterine fibroids.”

Twelve women (40.0%) described absenteeism or missing school, which included not attending work or school, arriving late, or leaving early from scheduled work or coursework because of UF symptoms. Women gave comments such as: “I wouldn't be able to go to work sometimes because I [would] be in so much pain,” and: “At school, I would just leave early because I did not feel good.” In addition, three women (10.0%) were unable to attend work or school for an extended period of time (*i.e.*, several months or years) because of UF symptoms, saying, for example: “Yes, because I haven't been working this whole year, pretty much because of my fibroids, because when I'm in pain, I'm in really bad pain.”

#### Financial burden

Almost half of women (*n* = 14; 46.7%) described a negative impact on finances. Eight women (*n* = 8 of 14; 57.1%) mentioned that this was a result of excessive spending on feminine hygiene, with one woman explaining: “I pretty much paid rent with the amount of money I was [spending on] buying for pads and tampons.” Other reasons included costs of missing work or school (*n* = 6 of 14; 42.9%), purchasing over-the-counter medications to cope with UF-associated symptoms (*n* = 3 of 14; 21.4%), replacing or cleaning soiled clothing (*n* = 2 of 14; 14.3%), and expenses related to physician visits (*n* = 1 of 14; 7.1%).

## Discussion

This study provides evidence of the high UF symptom burden on women with HMB associated with UF and the significant impacts that these symptoms can have on all aspects of life. As expected, all women with UF spontaneously mentioned experiencing HMB, which was an inclusion criterion of the LIBERTY studies. The women also reported experiencing different types of pain, with pelvic pain the most frequent. Tightness or pressure in the pelvic area was reported by about a quarter of women and described as different from pelvic pain. Although pain symptoms were more common during menstrual days, pain also occurred during nonmenstrual days. Pelvic pain was mainly experienced during menstrual days, while pressure in the pelvic area was reported on both menstrual and nonmenstrual days.

When asked about their symptom experience, women also mentioned experiencing anemia; it is likely that women used the medical term “anemia” in reference to a prior diagnosis by a health care provider. Women most commonly reported UF symptom impacts of feeling tired or fatigued, inability to complete daily activities, difficulty staying asleep, and a negative impact on social activities and their sex life.

Most published studies reporting UF symptoms and their impacts on women are cross-sectional in design and use survey techniques, whereas this research relied on interviews with open-ended questions, thereby allowing for exploration of all symptoms and impacts relevant to women. These methods enable the elicitation of women's descriptions and experiences in their own words, to provide a qualitative understanding of UF symptom and impact burden.

Currently, only one qualitative concept elicitation research study in UF has been published.^18^ The study, by Deal et al., was conducted in the United States in women with UF and comprised focus groups and cognitive interviews. It identified HMB, prolonged periods, pain, menstrual cramping, fatigue, and bloating as the most commonly reported symptoms in the focus groups. In individual cognitive interviews (*n* = 27), the most commonly mentioned UF symptoms were abnormal bleeding (*i.e.*, heavy, clots, prolonged, irregular), pain, and menstrual cramping. Pain symptoms included: back, abdominal, headache/migraine, leg, with ovulation, and pain during intercourse. The UF symptoms described in the present study are in accordance with those described by Deal et al.^18^ Fatigue was categorized as a UF symptom by these authors, whereas in the present study fatigue was considered to be a physical impact of UF, based on how women described it. Fatigue from a clinical perspective is a consequence of heavy blood loss and resultant anemia, suggesting that it is appropriate to consider this an impact.

Overall, the UF symptoms most commonly reported by women in this research (*i.e.*, HMB, pelvic pain, anemia, and urinary symptoms) are consistent with those presented in the literature. Symptoms described in the current study, but not in the literature, included hot flashes, chills, and weight gain; however, these were infrequently mentioned (*n* ≤ 5). Although not recorded in the current study, fertility problems have been reported as UF symptoms in reviews and in an interview study.^1,5,18^

The UF symptom impacts in this research are also generally consistent with those reported in the literature: negative impacts on emotional well-being, sex life, performance at work, daily activities, and energy levels.^5,19^ Similarly, the Uterine Bleeding and Pain Women's Research Study (Zimmermann et al.), which used a self-administered online survey for women with UF, reported that UF impacted on relationships and family, housekeeping, sport, and the type and color of clothes worn as a consequence of UF symptoms.^19^

Another impact of UF symptoms outlined in the present study, not previously reported in the literature, was financial strain and additional insights into the relationship between pain and menstruation assessed *post hoc*. Women reported that some symptoms were related exclusively to menstrual days, while other symptoms, primarily pain, were reported on nonmenstrual days also.

A possible limitation of this study was the inclusion of the Uterine Fibroid Symptom and Health Related Quality of Life Questionnaire (UFS-QoL) in LIBERTY 1 and 2 for completion at baseline and weeks 12 and 24 of the trials. The UFS-QoL is a UF-specific questionnaire that evaluates UF symptoms and their impact on quality of life.^20^ As a consequence of completing this questionnaire, women may have been primed to mention the symptoms and impacts assessed by this questionnaire, which may be a possible limitation of the study. To minimize this, women were encouraged to spontaneously report UF symptoms that were important to their lifetime experience of UF and were then given the opportunity to reflect.

In addition, while the sample size may be considered as small by some standards outside the realm of qualitative research, the size of this substudy proved to be sufficient based on the evaluation of saturation (*i.e.*, no new signs, symptoms, or impacts were reported after the 10th of the 30 interviews). The eligibility criteria required women to have HMB associated with UF; therefore, this study reports on the impact of UF in a population of women with confirmed HMB. Thus, the symptoms and impacts reported in this research may not be generalizable to women with UF not experiencing HMB. Prior research has shown that HMB is the most frequent symptom of UF, experienced by the majority of women with symptomatic UF.^3,21^

Furthermore, this study reports findings in a population of English-speaking women in the United States, which may limit generalizability of results. Further research on the burden of UF should include women experiencing any symptoms of UF, as well as women in countries other than the United States.

The strengths of the present study include the rich qualitative nature of the study data, which were collected in a sufficiently sized sample of women, as confirmed by the achievement of saturation. Women answered open-ended questions with minimal probing, giving women the opportunity to freely share their experiences and the burden that UF imposes on their lives. The demographic characteristics of women interviewed as part of the substudy were similar to those of the overall US LIBERTY 1 and 2 study populations (*N* = 582). Of note, the mean (standard deviation) age of women was 42.6 (4.6) and 42.1 (5.2) years in the substudy and overall US LIBERTY population, respectively, and the proportion of Black or African American patients was 70.0% and 63.9%, respectively.

## Conclusion

This interview study provides patient perspectives on symptoms experienced by women with HMB associated with UF and the negative impacts of these symptoms on women's daily life, described using their own words. After HMB, pain was the second most frequently mentioned symptom, including several different types of pain. Pain was most commonly experienced during menstrual days; however, some women also reported pain during nonmenstrual days. Symptoms impacted nearly every aspect of women's lives, including activities of daily life, work/school attendance and productivity, emotional well-being, sleep quality, and social activities, and placed a burden on women physically and financially. This study therefore provides further evidence of the significant UF burden on women with HMB associated with UF.

## Data Availability

Research data from the patient interviews that support the present analysis are not publically available.

## Abbreviations Used

HMB: heavy menstrual bleeding
PGA: patient global assessment
UF: Uterine fibroids
UFS-QoL: Uterine Fibroid Symptom and Health Related Quality of Life Questionnaire
